# Evolving Therapeutic Landscape of ROS1-Positive Non-Small Cell Lung Cancer: An Updated Review

**DOI:** 10.3390/curroncol32110626

**Published:** 2025-11-06

**Authors:** Hervé Bischoff, Sébastien Gendarme, Laura Somme, Christos Chouaid, Roland Schott

**Affiliations:** 1 Department of Medical Oncology, Institut de Cancérologie Strasbourg Europe, F-67200 Strasbourg, France; l.bender@icans.eu (L.S.); r.schott@icans.eu (R.S.); 2 Service de Pneumologie, Hôpital Tenon, APHP, Inserm U955, IMRB, Université Paris-Est Créteil, F-94010 Créteil, France; sebastien.gendarme@aphp.fr; 3 Centre de Recherche Clinique, CHI Créteil, Inserm U955, IMRB, Université Paris-Est Créteil, F-94010 Créteil, France; christos.chouaid@chicreteil.fr

**Keywords:** ROS1, non-small cell lung cancer, tyrosine kinase inhibitor, resistance mutations, brain metastases, targeted therapy, repotrectinib, taletrectinib, zidesamtinib

## Abstract

**Simple Summary:**

Lung cancer is the leading cause of cancer-related death worldwide. Among the different types, a small group of patients has tumors driven by changes in the *ROS1* gene. These cancers often spread to the brain and require treatments that can control the disease both in the body and the brain. The first drugs developed for ROS1-positive lung cancer, such as crizotinib, were effective but showed limitations over time, particularly with brain involvement and resistance. In recent years, several new medicines have been created to overcome these challenges. This article reviews the biology of ROS1-positive lung cancer. It provides an updated overview of the many treatments now available, including drugs that are more effective against resistance and that reach the brain more effectively. Understanding these advances is important for patients, doctors, and researchers, as they will shape future care and open new opportunities for clinical research.

**Abstract:**

*ROS1* gene rearrangements define a distinct molecular subtype of non-small cell lung cancer (NSCLC), occurring in approximately 2% of cases and frequently associated with younger age, non-smoker status, and a high incidence of brain metastases. The discovery of ROS1 as an oncogenic driver has led to the development of targeted tyrosine kinase inhibitors (TKIs). Crizotinib first demonstrated substantial clinical benefit, but its limitations, including poor central nervous system (CNS) penetration and acquired resistance, highlighted the need for next-generation inhibitors. Several agents have since been developed, including entrectinib, lorlatinib, repotrectinib, taletrectinib, and zidesamtinib, each offering improved intracranial (IC) activity and efficacy against resistance mutations, notably *ROS1^G2032R*. Despite these advances, optimal sequencing strategies remain undefined, and resistance ultimately emerges in most patients. This review provides an updated overview of ROS1 biology, diagnostic approaches, clinical outcomes with currently available TKIs, mechanisms of resistance, and ongoing challenges, emphasizing the rapidly evolving therapeutic landscape.

## 1. Introduction

Lung cancer remains the leading cause of cancer-related mortality worldwide [1]. An increasing proportion of newly diagnosed cases occur in never-smokers, a trend likely linked to the declining prevalence of tobacco use [2]. Among the molecular alterations identified in this population and in the search for new targetable oncogenic drivers following the success of tyrosine kinase inhibitors (TKIs) against EGFR, a chromosomal translocation involving the *ROS1* gene has been identified in the oncogenesis of lung cancer [3]. These fusions activate key oncogenic pathways and are associated with a higher incidence of brain metastases (BMs), highlighting the need for targeted therapies with both systemic and intracranial efficacy. Following the initial success of crizotinib, several next-generation ROS1 inhibitors have been developed to overcome resistance mutations and central nervous system (CNS) progression. This review is an update of the comprehensive review by Gendarme et al. [4], with a specific focus on the rapidly evolving therapeutic landscape of ROS1-positive non-small cell lung cancer (NSCLC).

## 2. ROS1 Biology

### 2.1. ROS1 Gene

*ROS1* encodes an orphan receptor tyrosine kinase belonging to the insulin receptor superfamily. A chromosomal rearrangement involving *ROS1* on chromosome 6 results in the formation of a constitutively active fusion protein that drives oncogenic transformation and activates downstream signaling pathways, including the MAPK, JAK/STAT, and PI3K cascades [5]. Phylogenetically, ROS1 is closely related to ALK, sharing 49% of its amino acid sequence [6].

The prevalence of *ROS1* rearrangements is estimated to be approximately 2% in NSCLC [7,8]. Dozens of fusion partners have been identified, among which *CD74*, *SLC34A2*, *EZR*, and *SDC4* are the most frequently reported [9]. The presence of a CD74–ROS1 fusion could be associated with prolonged survival outcomes compared with non-CD74–ROS1 rearrangements [10]. Co-mutations involving *EGFR*, *ALK*, or *KRAS* have been described but remain rare [11].

### 2.2. ROS1 Fusion Detection

ROS1 testing is recommended in all metastatic lung cancers, including mixed histologies where an adenocarcinoma component cannot be excluded, as well as in squamous-cell carcinomas occurring in never-smokers [12]. Screening for ROS1 fusions can be initiated using immunohistochemistry (IHC). All available ROS-1 antibody clones show high sensitivity (90–100%), while their specificity remains more variable (70–90%) [13]. Due to a high rate of false positives, particularly in acinar or lepidic NSCLC and in EGFR-mutated cases [14], any positive result must be confirmed by in situ hybridization (ISH) [12].

ISH remains the reference method, capable of detecting *ROS1* rearrangements regardless of the fusion partner, although it does not allow partner identification. Reliable assessment requires sufficient tumor cellularity, and both false positives and false negatives may occur, especially with rare fusion variants. The technique remains costly and time-consuming. Dual ALK/ROS1 break-apart probe kits can reduce tissue requirements and allow testing on cytological samples [15]. A strategy combining IHC for initial screening and ISH for confirmation is considered the most efficient approach in terms of turnaround time, cost-effectiveness, and positive predictive value [16].

Molecular biology techniques such as reverse transcription PCR (RT-PCR) and next-generation sequencing (NGS) are increasingly being adopted, as they provide high sensitivity and can detect rare or novel fusions as well as other actionable alterations. However, RT-PCR is technically complex and prone to variability, and NGS—although comprehensive—remains limited in routine practice by its cost, longer turnaround time, and occasional discordance with IHC or ISH [12]. Finally, from a public payer perspective, systematic *ROS1* testing has been demonstrated to be cost-effective [17].

## 3. Epidemiology

Compared to NSCLC without an oncogenic driver mutation, the prevalence of ROS1-mutated NSCLC is higher among women, younger patients, non-smokers, and Asian populations [8]. Adenocarcinomas are the predominant histological subtype. Clinically, 75–85% of patients present with metastatic disease at diagnosis [8,18], and BMs are already present at diagnosis in approximately 30% to 40% of cases [19]. Pericardial and lymph node metastases are also more frequently observed compared to other NSCLCs harboring oncogenic drivers [20]. Prognosis is particularly influenced by the development and progression of BMs, with a cumulative incidence reported as high as 75% [19].

## 4. Efficacy of Conventional Treatments

The efficacy of pemetrexed-based chemotherapy has been reported in patients with ROS1-rearranged tumors [21]. Compared to other oncogenic drivers such as ALK, EGFR, and KRAS, patients with ROS1 fusion–positive tumors exhibit higher overall response rates (ORR) and longer progression-free survival (PFS) when treated with pemetrexed-based regimens [22]. However, most available data come from studies using platinum-pemetrexed doublets, making it difficult to determine whether this benefit reflects intrinsic chemosensitivity or a specific sensitivity to pemetrexed. In the retrospective European EUROS1 cohort, the ORR to chemotherapy, predominantly pemetrexed-based, was 58%, with a median PFS of 7.2 months [23].

The efficacy of immune checkpoint inhibitors (ICIs) appears to be limited in patients harboring oncogenic drivers such as EGFR and ALK [24,25]. Data in ROS1-rearranged NSCLC are also limited and largely retrospective. Although some studies have reported clinical benefit with ICIs in ROS1-positive patients [26], the overall activity of ICI monotherapy remains modest in this setting, despite higher PD-L1 expression frequently observed in ROS1-rearranged tumors [27]. Chemo-immunotherapy combinations can achieve more meaningful responses (median time to treatment discontinuation 10 months, ORR 83%) [28]; however, this benefit is likely driven largely by the chemotherapy backbone rather than the immunotherapy component. Across retrospective series, findings remain inconsistent and limited by small sample sizes. Current guidelines recommend ROS1-directed TKIs as first-line therapy. Immunotherapy monotherapy is not recommended in ROS1-rearranged NSCLC, and chemo-immunotherapy is not specifically endorsed, with chemotherapy remaining the preferred option when targeted or local strategies are not appropriate.

## 5. Treatment of ROS-1-Positive NSCLCs

### 5.1. Crizotinib

Crizotinib is an oral TKI initially developed to target ALK and MET receptors, which was later found to also inhibit fusion proteins involving ROS1. Its efficacy in ROS1-rearranged NSCLC was primarily demonstrated in the PROFILE 1001 study, a phase I trial with a dedicated cohort expansion. In this cohort, which included 53 patients with advanced ROS1-positive NSCLC, crizotinib achieved an ORR of 72% (95% CI 58–83), including 6 complete responses and 32 partial responses [29]. Responses were rapid (median time to response, 7.9 weeks) and durable, with a median duration of response (DOR) of 24.7 months (95% CI 15.2–45.3). The median PFS was 19.3 months (95% CI 15.2–45.3), and the median overall survival (OS) was estimated at 51.4 (95% CI 29.3–not reached (NR)) months, with a 4-year survival probability of 51% [30]. These results were consistent across different fusion partners, suggesting that the clinical benefit of crizotinib is independent of the specific *ROS1* rearrangement [30].

Treatment-related adverse events (AEs) were mostly grade 1 or 2, with the most frequent being visual disturbances (82%), nausea (40%), peripheral edema (40%), and diarrhea (44%). Grade ≥ 3 toxicities were rare, primarily hypophosphatemia and neutropenia. No AEs led to permanent treatment discontinuation, and no grade 4 or 5 events were reported.

The results of PROFILE 1001 were supported by other prospective studies. A phase II trial conducted in Asia involving 127 patients reported a similar ORR of 72% (95% CI 63–79) and a median PFS of 15.9 months [31]. A French study (AcSé) also demonstrated comparable activity (ORR 69.4%, 95% CI 53–82), although with a shorter median PFS of 5.5 (95% CI 4.2–9.1) months, likely reflecting poorer baseline performance status [32]. Similarly, the European phase II EUCROSS trial, which enrolled 34 patients, reported an ORR of 70% (95% CI 51–85) and a median PFS of 20 (95% CI 8–NR) months [33]. Consistent findings were also observed in the Italian METROS study [34].

Crizotinib thus established a new standard of care for patients with *ROS1*-rearranged NSCLC, leading to its approval by both the FDA and the EMA in 2016.

### 5.2. Entrectinib

Although crizotinib demonstrates systemic efficacy, it has limited penetration into the CNS and does not prevent CNS progression, which is common in ROS1-positive NSCLC. To address this limitation, entrectinib was developed as a TKI capable of crossing the blood–brain barrier, providing both systemic and intracranial activity. Entrectinib specifically targets ROS1, NTRK, and ALK and was designed to efficiently penetrate the blood–brain barrier [35].

In an updated integrated analysis of the ALKA-372-001, STARTRK-1, and STARTRK-2 trials, which included 168 TKI-naïve patients with advanced ROS1-positive NSCLC, entrectinib demonstrated durable systemic and intracranial efficacy [36]. The ORR was 68% (95% CI 60.2–74.8), including 13% complete responses, with a median DOR of 20.5 (95% CI 14.8–34.8) months and a median PFS of 15.7 (95% CI 12.0–21.1) months. The median OS was 47.8 (95% CI 44.1–NR) months, comparable to that reported with crizotinib [36]. Among patients with baseline BMs (*n* = 48), intracranial activity was notable, with an intracranial ORR of 80% (95% CI 59.3–93.2) among those with measurable disease, and a median intracranial DOR of 12.9 months (95% CI 7.1–22.1).

A specific cohort of patients who had developed isolated intracranial progression on crizotinib (*n* = 18) was also analyzed. In this setting, entrectinib demonstrated more modest efficacy, with a global ORR of 11% (95% CI 1.4–34.7), an intracranial ORR of 19% (95% CI 4.1–45.7), and a median PFS of 4.7 (95% CI 2.9–43.5) months, suggesting limited benefit after CNS progression on crizotinib, potentially due to underlying resistance mechanisms.

The safety profile of entrectinib was characterized by a distinct pattern of neurological adverse events, including dysgeusia, dizziness, and paresthesia, likely related to the inhibition of TRK family tyrosine kinase receptors involved in synaptic control and plasticity [37]. Metabolic toxicities, such as weight gain and elevated creatinine levels, were also observed but rarely led to treatment discontinuation.

Entrectinib subsequently became an approved treatment option for patients with *ROS1*-rearranged NSCLC, receiving FDA approval in 2019 and EMA approval in 2020, based on its demonstrated systemic and intracranial efficacy. A phase III trial (NCT04603807) is ongoing to compare entrectinib with crizotinib in a TKI-naïve population.

### 5.3. Ceritinib

Ceritinib is an oral TKI initially developed for patients with ALK-positive NSCLC but also demonstrated activity against *ROS1* rearrangements due to the structural homology between these two kinases. Its efficacy was evaluated in a prospective multicenter Korean study [38] that included 32 patients with ROS1-positive NSCLC. Most patients (*n* = 30) were TKI-naïve but had received multiple lines of chemotherapy (median: 3 lines). The ORR was 62% (95% CI 45–77), with a median DOR of 21 (95% CI 17–25) months. Among crizotinib-naïve patients, the ORR reached 67% (95% CI 48–81), and the median PFS was 19.3 months (95% CI 1–37), figures comparable to those observed with crizotinib in the PROFILE 1001 study. The median OS in this population was 24 months (95% CI 5–43).

Regarding intracranial activity, data from this study remains limited but encouraging: among the eight patients with baseline BMs, a CNS disease control rate of 63% (95% CI 31–86) was observed, with two patients achieving an objective intracranial response. Although based on a small sample size, these findings suggest a degree of ceritinib efficacy in the CNS compartment, particularly in the absence of prior radiotherapy. These observations are supported by results from the ASCEND-1 and ASCEND-2 trials conducted in ALK-positive patients, where ceritinib demonstrated significant penetration across the blood–brain barrier [39].

In terms of safety, the most frequently observed AEs with ceritinib were gastrointestinal (diarrhea 85%, nausea 72%, vomiting 45%, anorexia 56%), predominantly grade 1–2. Dose reductions were required in 68% of patients, without major impact on treatment efficacy. However, the safety profile remains more challenging than that of newer-generation inhibitors, particularly with long-term use.

Ceritinib represents a therapeutic option for ROS1-positive NSCLC, especially in crizotinib-naïve patients. It shows systemic efficacy comparable to crizotinib and potential intracranial activity that warrants further confirmation, although its clinical development in this indication has been limited by the emergence of newer, more selective, and better-tolerated inhibitors. It is important to note that ceritinib does not have formal regulatory approval for ROS1-rearranged NSCLC in any region.

### 5.4. Lorlatinib

Lorlatinib is a novel generation oral TKI designed to selectively target ROS1 and ALK, with demonstrated ability to cross the blood–brain barrier by reducing the efflux mediated by P-glycoprotein-1. A prospective phase I/II study enrolled 69 patients with advanced ROS1-positive NSCLC, including 21 TKI-naïve patients and 40 patients previously treated with crizotinib [40]. Among TKI-naïve patients, the ORR was 62% (95% CI 38–82), with a median DOR of 25.3 (95% CI 7.5–31.9) months and a median PFS of 21.0 (95% CI 4.2–31.9) months. Notably, significant intracranial activity was also reported, with an intracranial ORR of 64% (95% CI 31–89) among patients with BMs at baseline [40].

In patients previously treated with crizotinib, lorlatinib demonstrated a more modest ORR (35%, 95% CI 21–52) but achieved durable responses (median DOR of 13.8 months, 95% CI 9.7–NR) and meaningful intracranial disease control (intracranial ORR of 50%, 95% CI 25–75). Interestingly, lorlatinib showed activity in some patients harboring secondary resistance mutations to crizotinib, notably *ROS1^K1991E* and *ROS1^S1986F* mutations, but exhibited limited efficacy against the commonly observed *ROS1^G2032R* mutation [40].

These findings were confirmed by a phase II study conducted in South Korea, dedicated to TKI-naïve ROS1-positive patients. Among the 32 patients enrolled, 66% of whom were receiving first-line treatment, the ORR reached 69% (95% CI 52–83), with a median PFS of 35.8 (95% CI NR–NR) months and an overall favorable safety profile [41].

The French real-world LORLATU study [42] provided complementary data on 80 patients treated with lorlatinib after at least one prior ROS1-targeted TKI. The ORR was 45% (95% CI not provided), with an intracranial ORR of 72% (95% CI not provided), a median PFS of 7.1 (95% CI 5.0–9.9) months, and a median OS of 19.6 (95% CI 12.3–27.5) months. Among patients with non-irradiated brain metastases, the median intracranial DOR reached 20.6 (95% CI 1.9–22.7) months. These results highlight the sustained efficacy of lorlatinib in a more heterogeneous, heavily pretreated, and real-world patient population.

The most common AEs associated with lorlatinib were neurocognitive (mood alterations, dizziness, mostly grade 1–2) and metabolic disorders, with grade 3–4 events such as hypertriglyceridemia and hypercholesterolemia also reported [40]. Treatment discontinuation due to AEs occurred in only 1% of patients in the pivotal trial [40], compared to 13% in the real-world LORLATU cohort [42].

Furthermore, mechanisms of resistance to lorlatinib have been explored in patients progressing under crizotinib or lorlatinib [43]. ROS1 mutations were identified in 38% of biopsies post-crizotinib and in 46% post-lorlatinib, with *ROS1^G2032R* being the most frequently observed. Novel mutations, such as *ROS1^L2086F*, either alone or in combination with G2032R or S1986F, were also identified following progression on lorlatinib. Preclinical models suggest that these mutations confer cross-resistance to crizotinib, entrectinib, and lorlatinib, while potentially retaining sensitivity to cabozantinib [44]. In addition, ROS1-independent resistance mechanisms have been described, including *MET* and *KRAS* amplifications as well as *NRAS* and *MAP2K1* mutations [43].

Despite the demonstrated efficacy of lorlatinib, particularly against BMs and certain secondary mutations, resistance remains common, especially in the presence of *ROS1^G2032R* and *ROS1^L2086F* mutations. In this context, the development of next-generation inhibitors with optimized structures, such as repotrectinib, aims to overcome these resistance mechanisms.

### 5.5. Repotrectinib

Repotrectinib is a next-generation oral TKI designed to target ROS1, ALK, and NTRK rearrangements, including those associated with resistance mutations, notably solvent front substitutions such as *ROS1^G2032R* and D2033N. Its compact macrocyclic structure reduces steric hindrance and improves penetration into the CNS [45].

The clinical efficacy of repotrectinib was evaluated in the phase I/II TRIDENT-1 study [46], which enrolled 127 patients with advanced *ROS1*-rearranged NSCLC. Among ROS1 TKI-naïve patients (*n* = 71), the ORR was 79% (95% CI 68–88), with a median DOR of 34.1 months (95% CI 25.6–NR) and a median PFS of 35.7 months (95% CI 27.4–NR). Intracranial activity was also notable, with an intracranial response rate of 89% (95% CI 52–100) among patients with measurable BMs at baseline.

In patients previously treated with a ROS1-targeted TKI (*n* = 56), repotrectinib maintained meaningful activity, with an ORR of 38% (95% CI 25–52) and a median PFS of 9 months (95% CI 6.8–19.6). Notably, in a subset of 17 patients harboring the *ROS1^G2032R* resistance mutation, a response rate of 59% (95% CI 33–82) was observed, underscoring repotrectinib’s ability to overcome specific resistance mechanisms [46]. Preclinical data had already demonstrated the potent inhibitory activity of repotrectinib against *ROS1^G2032R* and D2033N mutations, outperforming lorlatinib and entrectinib in vitro [45].

In parallel, the safety profile was favorable, with most AEs being grade 1–2, predominantly dizziness (58%), dysgeusia (50%), and paresthesia (30%), leading to treatment discontinuation in 7% of cases [46]. Altogether, these findings position repotrectinib as a reference inhibitor for patients with ROS1-positive NSCLC, particularly in the presence of resistance mutations or CNS involvement, representing a major advance over first-generation inhibitors.

Repotrectinib received FDA approval in 2023 and conditional EMA approval in 2024, based on its demonstrated efficacy against resistance mutations and CNS metastases. A phase III trial (NCT06140836) is ongoing to compare repotrectinib with crizotinib in a TKI-naïve population.

### 5.6. Taletrectinib

Taletrectinib is a next-generation oral TKI, highly selective for ROS1, designed to overcome several limitations associated with first-generation inhibitors such as crizotinib and entrectinib: limited CNS penetration, neurological toxicity related to TRK inhibition, and inefficacy against certain resistance mutations, particularly *ROS1^G2032R* [47]. With a selectivity for ROS1 that is 11 to 20 times greater than for TRK, taletrectinib promises improved neurological tolerability and enhanced intracranial activity [47].

The recently published integrated analysis of the TRUST-I [48] and TRUST-II [47] trials evaluated the efficacy and safety of taletrectinib in 273 patients with ROS1-positive NSCLC [49,50]. Among TKI-naïve patients (*n* = 160), the confirmed ORR was 88.8% (95% CI 82.8–93.2), with a median DOR of 44.2 (95% CI 30.4–NR) months and a median PFS of 45.6 (95% CI 29.0–NR) months [50]. The intracranial ORR among patients with measurable BMs was 76.5% (95% CI 50.1–93.2), with a median intracranial DOR of 14.7 (95% CI 4.2–30.2) months [50].

In patients previously treated with a ROS1 TKI (*n* = 113, including 91% with crizotinib and 9% with entrectinib), the ORR was 55.8% (95% CI 46.1–65.1), with a median DOR of 16.6 (95% CI 10.6–27.3) months and a median PFS of 9.7 (95% CI 7.4–12.0) months [50]. Intracranial activity was also notable, with an intracranial ORR of 65.6% (95% CI 46.8–81.4). Among the 13 patients harboring the *ROS1^G2032R* resistance mutation, an objective response was observed in 8 patients (61.5%, 95% CI 31.6–86.1), confirming substantial activity against this frequent resistance mutation, comparable to that observed with repotrectinib [50].

The safety profile was overall favorable. The most common AEs were gastrointestinal in the first days (diarrhea 61% and nausea 44%) and biological (transaminase elevation 70%), predominantly grade 1. Neurological events (dizziness 21%, dysgeusia 15%) were infrequent and generally mild [47,48,50]. The rate of treatment discontinuation due to toxicity was 6.5% [50].

Comparison between and within Asian regional TRUST-I and global TRUST-II showed similar efficacy and safety profile across Asian and Western populations [51], confirming the broad applicability of taletrectinib’s clinical benefit. A phase III trial (NCT06564324, TRUST-III) is ongoing to compare taletrectinib with crizotinib in a TKI-naïve population. Nevertheless, the consolidated data from TRUST-I and TRUST-II already position taletrectinib as one of the most promising agents in the ROS1 inhibitor class, offering sustained efficacy, documented CNS penetration, activity against resistance mutations, and a favorable tolerability profile [50].

### 5.7. Zidesamtinib

Zidesamtinib (NVL-520) is a next-generation oral TKI specifically designed to overcome the limitations of previous TKIs in ROS1 fusion–positive cancers. It is distinguished by its unique triple pharmacological specificity: potent activity against ROS1 and its resistance mutations (notably G2032R), selective sparing of TRK kinases often implicated in neurological AEs, and excellent CNS penetration [52].

In preclinical studies, NVL-520 demonstrated subnanomolar inhibitory activity against wild-type ROS1 and retained remarkable potency against the *ROS1^G2032R* mutation (IC_50_ = 7.9 nM), outperforming crizotinib, entrectinib, lorlatinib, and even repotrectinib in this context [52]. This selectivity is attributed to its macrocyclic structure, which minimizes steric hindrance at the G2032R binding site while optimizing interactions with the ROS1 catalytic pocket, all while avoiding TRK kinase inhibition.

Early clinical results from the phase I/II ARROS-1 study, presented at ESMO 2024 [53] and WCLC 2025 (World Conference on Lung Cancer; 6–9 September 2025; Barcelona, Spain. Abstract 4540, not yet published), confirm these preclinical promises. Among the 117 efficacy-evaluable TKI-pretreated patients, the median number of prior therapies was 2, with half having received at least 2 prior ROS1 TKIs and 93% exposed to lorlatinib, repotrectinib, and/or taletrectinib. CNS involvement was present in 49% of patients. Durable responses were achieved even in patients with CNS disease, the *ROS1^G2032R* mutation, or multiple prior ROS1 TKIs. In this pretreated population, the ORR was 44% (95% CI 34–53) with a 12-month DOR rate of 78%. Patients who had only received crizotinib or entrectinib with or without chemotherapy (*n* = 55) achieved an ORR of 51% (95% CI 37–65) and an 18-month DOR rate of 93%. Preliminary results in TKI-naïve patients (*n* = 35) were particularly striking, with an ORR of 89% and a 12-month DOR rate of 96% (95% IC not provided). Intracranial activity was notable, with an IC-ORR of 83%, including four complete responses, and no CNS progression events at data cutoff.

Zidesamtinib was generally well tolerated, with the most common AEs being peripheral edema (36%), constipation (17%), creatine phosphokinase elevation (16%), fatigue (16%), and dyspnea (15%). Dose reductions occurred in 10% of patients, and treatment discontinuations in 2% (most common reason: pneumonia). Thus, zidesamtinib emerges as a highly promising candidate for overcoming resistance, particularly in cases involving the G2032R mutation or CNS involvement. Its TRK-sparing profile confers improved neurological tolerability. As its development progresses, zidesamtinib could reshape the therapeutic landscape of ROS1-positive NSCLC by addressing the two major challenges of this disease: resistance mutations and CNS dissemination. A rolling NDA submission is expected to be completed in Q3 2025, with ongoing discussions with the FDA regarding potential line-agnostic indications [54].

## 6. Mechanisms of Resistance

**Figure 1:**
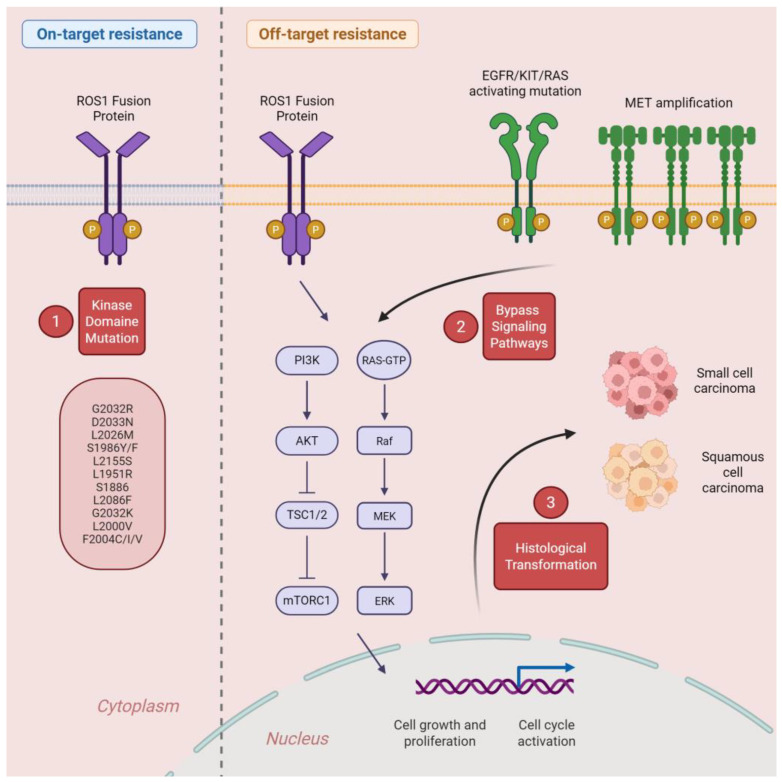
Mechanisms of On-target and Off-target Resistance to ROS1 Inhibitors in NSCLC. Schematic representation of downstream signaling pathways activated by ROS1 fusions and the main mechanisms of acquired resistance to ROS1-targeted TKIs. On-target resistance involves secondary mutations within the ROS1 kinase domain (e.g., G2032R, D2033N, L2086F, L2026M, S1986F) that reduce drug binding affinity. Off-target resistance includes activation of bypass signaling through amplification or mutations in alternative oncogenic drivers such as EGFR, MET, KIT, KRAS, NRAS, and MAP2K1, leading to reactivation of the MAPK and PI3K/AKT/mTOR pathways. These adaptive changes allow tumor cells to maintain proliferation and survival despite continued ROS1 inhibition. Abbreviations: ROS1: c-ros oncogene 1 receptor tyrosine kinase; EGFR: Epidermal Growth Factor Receptor; KIT: KIT proto-oncogene receptor tyrosine kinase; RAS: Rat Sarcoma viral oncogene homolog; PI3K: Phosphatidylinositol-3-kinase; AKT: Protein kinase B; TSC1/2: Tuberous Sclerosis Complex 1/2; mTORC1: Mechanistic Target of Rapamycin Complex 1; RAS-GTP: Active GTP-bound form of RAS; Raf: Rapidly Accelerated Fibrosarcoma kinase; MEK: Mitogen-Activated Protein Kinase; ERK: Extracellular Signal-Regulated Kinase; MET: Mesenchymal–Epithelial Transition factor; NSCLC: Non-Small Cell Lung Cancer. Created in BioRender. Bischoff, H. (2025) https://BioRender.com/kl23zg5 (accessed on 2 November 2025).

Despite the initial effectiveness of ROS1-targeted TKIs, acquired resistance inevitably develops, leading to disease progression. Resistance mechanisms can be broadly categorized into two groups: on-target and off-target alterations (Figure 1).

On-target resistance (approx. 40%) involves secondary mutations within the ROS1 kinase domain that impair TKI binding [43,55,56,57]. The most clinically relevant is *ROS1^G2032R* [57], a solvent-front substitution that confers high-level resistance to crizotinib, entrectinib, and lorlatinib, while maintaining some sensitivity to newer agents such as repotrectinib, taletrectinib, and zidesamtinib [44]. Other important mutations include D2033N, L2086F, L2026M, and S1986F, each associated with variable patterns of cross-resistance depending on the TKI used [58]. Notably, L2086F is particularly challenging, as it is located deeper within the ATP-binding pocket. This alteration is associated with cross-resistance to many existing TKIs, including repotrectinib, taletrectinib, and zidesamtinib. In such cases, multitargeted TKIs such as cabozantinib or gilteritinib, which display off-target ROS1 inhibitory activity, have demonstrated preclinical efficacy and could represent salvage options [44].

Off-target resistance mechanisms (approx. 60%) encompass genetic alterations that bypass ROS1 inhibition, notably *MET* amplification [59], *KRAS* mutations or amplifications, *NRAS* mutations [60], *KIT* mutation [61], and *MAP2K1* mutations [62]. These bypass tracks activate alternative oncogenic pathways, sustaining tumor growth independently of ROS1 signaling. Molecular profiling at progression, including rebiopsy or liquid biopsy, is essential to adapt therapeutic strategies accordingly. Histologic transformation, although less common, has also been described as an off-target resistance mechanism, notably including transitions to small-cell lung cancer or squamous histology [63]. As seen in ALK-rearranged NSCLC, the adoption of next-generation TKIs in the first-line setting shifts the resistance landscape toward fewer on-target events and a greater proportion of off-target mechanisms, including histologic transformation [64]. A comparable pattern is likely to emerge in ROS1-rearranged disease as newer inhibitors move to the front.

## 7. Therapeutic Strategies and Future Directions

Table 1 provides a comparative overview of efficacy outcomes with evaluated ROS1 TKIs in the first-line setting, highlighting differences in systemic and intracranial activity. If access permits, repotrectinib and taletrectinib both appear as highly attractive options. However, these observations should be interpreted with caution, as cross-trial comparisons have inherent limitations and definitive conclusions will require head-to-head randomized studies. Repotrectinib demonstrates strong activity against solvent front mutations and offers robust intracranial control, whereas taletrectinib shows similarly impressive systemic and CNS efficacy, with an even lower rate of neurological AEs due to its TRK-sparing profile.

The choice between these agents may ultimately depend on specific patient characteristics, including baseline CNS involvement, expected tolerance, and local drug availability. Table 2 summarizes the safety profile of available ROS1 TKIs, highlighting differences in notable and grade ≥ 3 adverse events that may influence treatment selection. Pending results from ongoing phase III trials, these next-generation inhibitors may replace crizotinib as the standard initial treatment.

With disease progression, prognosis generally worsens as therapeutic pressure promotes the emergence of resistant subclones. Subsequent management depends on the pattern of relapse. For patients receiving a first-line ROS1-targeted TKI such as repotrectinib, entrectinib, crizotinib, or ceritinib, CNS progression should prompt local treatment strategies such as radiation, ablation, or surgery with continuation of the current TKI in cases of oligoprogression or alternatively switching to a ROS1 inhibitor with superior CNS penetration, including entrectinib, lorlatinib, repotrectinib, taletrectinib, or zidesamtinib. In cases of systemic progression outside the CNS, biomarker testing using liquid biopsy or tumor tissue biopsy should be performed. If a ROS1 resistance mutation is detected, treatment with a TKI active against acquired mutations, such as repotrectinib, taletrectinib, zidesamtinib, or lorlatinib, is recommended. Table 3 summarizes the clinical activity of next-generation ROS1 TKIs in pretreated patients, including intracranial outcomes and efficacy against the G2032R mutation.

If no ROS1 resistance mutation is found, it is advisable to reassess ROS1 dependence, verify the presence of alternative or additional oncogenic drivers, and adapt systemic treatment accordingly, including rational therapeutic combinations, pemetrexed-based chemotherapy if chemotherapy-naïve, or participation in clinical trials. In cases of oligoprogression outside the CNS, local ablative therapies followed by continuation of the same TKI remain appropriate options.

Optimal sequencing strategies after failure of first-line TKIs are not yet well defined. The role of immunotherapy, either alone or in combination with targeted therapies, remains to be elucidated in ROS1-positive NSCLC. Additionally, head-to-head comparisons between new-generation inhibitors are lacking, making it difficult to establish the best therapeutic approach based solely on current evidence. A selection of ongoing clinical trials is summarized in Table 4.

Although evidence in ROS1-rearranged NSCLC predominantly derives from the metastatic setting, interest in peri-operative ROS1 inhibition is emerging. Case reports and small series have described the feasibility of (neo)adjuvant TKIs [65,66,67,68]. Prospective trials are now underway, including a single-arm neoadjuvant study of WX-0593 (NCT05765877), the phase III TRUST-IV trial evaluating adjuvant taletrectinib versus placebo (NCT07154706), and the umbrella trials UPLIFT (NCT06955325) and NAUTIKA1 (NCT04302025). A similar strategy has already been validated in ALK-rearranged NSCLC, where the phase III ALINA trial established adjuvant alectinib as a standard of care, supporting a comparable evolution for ROS1-targeted therapy as randomized peri-operative data mature [69]. High-level evidence remains awaited before routine use in the curative-intent setting.

## 8. Conclusions

The therapeutic landscape of ROS1-positive NSCLC is rapidly evolving. The development of highly selective, brain-penetrant inhibitors capable of overcoming resistance mutations offers new hope for durable disease control. Future research should prioritize biomarker-driven sequencing strategies and head-to-head comparisons of next-generation inhibitors to optimize long-term patient outcomes.

## Figures and Tables

**Table 1:** Efficacy outcomes of ROS1 tyrosine kinase inhibitors in TKI-naïve patients with ROS1-positive NSCLC, including intracranial activity when available.

Drug [Reference]	Targets	*n*	TKI-Naive ORR (%)	mPFS (Months)	IC-ORR (%)
Crizotinib [29]	ROS1, ALK, MET	50	72	19.2	/
Entrectinib [36]	ROS1, TRK, ALK	168	68	15.7	80 ^a^
Ceritinib [38]	ROS1, ALK	30	67	19.3	25 ^b^
Lorlatinib [40]	ROS1, ALK	21	62	21.0	64 ^c^
Repotrectinib [46]	ROS1, TRK, ALK	71	79	35.7	89 ^d^
Taletrectinib [50]	ROS1 (TRK-sparing)	160	88.8	45.6	76.5 ^e^

Patients evaluable for intracranial ORR: *n* = 25 ^a^; *n* = 8 ^b^; *n* = 11 ^c^; *n* = 9 ^d^; *n* = 17 ^e^. Abbreviations: ALK, anaplastic lymphoma kinase; IC, intracranial; mPFS, median progression-free survival; MET, mesenchymal–epithelial transition factor; NSCLC, non-small cell lung cancer; ORR, overall response rate; ROS1, c-ros oncogene 1; TRK, tropomyosin receptor kinase; TKI, tyrosine kinase inhibitor.

**Table 2:** Notable adverse events, main grade ≥ 3 toxicities, and discontinuation rates associated with ROS1 tyrosine kinase inhibitors in ROS1-positive NSCLC.

Drug [Reference]	Notable AEs	Main Grade ≥ 3 AEs (%)	Discontinuation for AEs (%)
Crizotinib [29]	Visual disturbances (82%), diarrhea (44%), nausea (40%), peripheral edema (40%)	Hypophosphatemia (10%), neutropenia (10%)	<1
Entrectinib [36]	Dysgeusia (40%), dizziness (37%), constipation (32%), paresthesia (18%)	Weight gain (11%)	5
Lorlatinib [40]	Hypercholesterolemia (80%), cognitive effects (27%), mood disorders (16%), dizziness (12%), weight gain (23%)	Hypercholesterolemia (15%), hypertriglyceridemia (19%), weight gain (7%)	1
Repotrectinib [46]	Dizziness (58%), dysgeusia (50%), paresthesia (30%)	Dizziness (3%), anemia (4%)	7
Taletrectinib [50]	Increased AST/ALT (70%), diarrhea (61%), nausea (44%), vomiting (41%), dizziness (15%), QT prolongation (18%)	Increased AST/ALT (9%), diarrhea (3%), anemia (3%), QT prolongation (3%), neutropenia (4%)	2
Zidesamtinib ^a^	Peripheral edema (36%), constipation (17%), CPK elevation (16%), fatigue (16%), dyspnea (15%)	CPK elevation (3%), dyspnea (3%)	2

^a^ Reference: World Conference on Lung Cancer; 6–9 September 2025; Barcelona, Spain. Abstract 4540. Abbreviations: AE, adverse event; ALT, alanine aminotransferase; AST, aspartate aminotransferase; CPK, creatine phosphokinase; QT, QT interval on electrocardiogram.

**Table 3:** Clinical outcomes of next-generation ROS1 tyrosine kinase inhibitors in TKI-pretreated patients, including intracranial efficacy and activity against the G2032R resistance mutation.

Drug [Reference]	Targets	*n*	TKI-Pretreated ORR (%)	mPFS (Months)	IC-ORR (%)	ORR if G2032R Mutation (%)
Lorlatinib [40]	ROS1, ALK	40	62	8.5	50	0
Repotrectinib [46]	ROS1, TRK, ALK	56	38	9.0	38	59
Taletrectinib [50]	ROS1 (TRK-sparing)	113	55.8	9.7	65.6	67
Zidesamtinib ^a^	ROS1 (TRK-sparing)	117	44	9.7	48	83

^a^ Reference: World Conference on Lung Cancer; 6–9 September 2025; Barcelona, Spain. Abstract 4540. Abbreviations: ALK, anaplastic lymphoma kinase; G2032R, solvent-front resistance mutation in *ROS1*; IC, intracranial; mPFS, median progression-free survival; ORR, overall response rate; *ROS1*, c-ros oncogene 1; TRK, tropomyosin receptor kinase; TKI, tyrosine kinase inhibitor.

**Table 4:** Selection of ongoing clinical trials investigating ROS1-targeted tyrosine kinase inhibitors in ROS1-positive NSCLC.

Trial	Population	Phase	Planned Enrollment (*n*)	Study Drugs	Primary Endpoint	Status
NCT06564324 (TRUST-III)	TKI-naive	III	138	Taletrectinib vs. Crizotinib	PFS	Recruiting
NCT06140836 (TRIDENT-3)	TKI-naive	III	230	Repotrectinib vs. Crizotinib	PFS	Recruiting
NCT04603807 (MO41552)	TKI-naive	III	220	Entrectinib vs. Crizotinib	PFS	Recruiting
NCT04621188 (ALBATROS)	TKI-pretreated	II	54	Lorlatinib (single arm)	ORR	Active, not recruiting
NCT05297890	TKI-pretreated	II	70	Lorlatinib (single arm)	ORR	Active, not recruiting
NCT06128148	NR	I	54	JYP0322 (single arm)	DLT	Recruiting

Abbreviations: DLT, dose-limiting toxicity; MTD, maximum tolerated dose; NSCLC, non-small cell lung cancer; ORR, overall response rate; PFS, progression-free survival; RP2D, recommended phase 2 dose; TKI, tyrosine kinase inhibitor.

## Data Availability

No new data were created or analyzed in this study.

## Abbreviations

The following abbreviations are used in this manuscript:

AE	Adverse event
ALK	Anaplastic lymphoma kinase
ALT	Alanine aminotransferase
AST	Aspartate aminotransferase
BMs	Brain metastases
CI	Confidence interval
CNS	Central nervous system
CPK	Creatine phosphokinase
DLT	Dose-limiting toxicity
DOR	Duration of response
EGFR	Epidermal growth factor receptor
EMA	European Medicines Agency
FDA	Food and Drug Administration
IC	Intracranial
IC-ORR	Intracranial overall response rate
ICI	Immune checkpoint inhibitor
IHC	Immunohistochemistry
ISH	In situ hybridization
MAPK	Mitogen-activated protein kinase
MET	Mesenchymal–epithelial transition factor
mPFS	Median progression-free survival
NGS	Next-generation sequencing
NR	Not reached
NSCLC	Non-small cell lung cancer
ORR	Overall response rate
OS	Overall survival
PCR	Polymerase chain reaction
PD-L1	Programmed death-ligand 1
PFS	Progression-free survival
QT	QT interval on electrocardiogram
RT-PCR	Reverse transcription polymerase chain reaction
TKI	Tyrosine kinase inhibitor
TRK	Tropomyosin receptor kinase
